# Deriving spatial features from *in situ* proteomics imaging to enhance cancer survival analysis

**DOI:** 10.1093/bioinformatics/btad245

**Published:** 2023-06-30

**Authors:** Monica T Dayao, Alexandro Trevino, Honesty Kim, Matthew Ruffalo, H Blaize D’Angio, Ryan Preska, Umamaheswar Duvvuri, Aaron T Mayer, Ziv Bar-Joseph

**Affiliations:** Joint Carnegie Mellon University—University of Pittsburgh Ph.D. Program in Computational Biology, Pittsburgh, PA 15213, United States; Computational Biology Department, Carnegie Mellon University, Pittsburgh, PA 15213, United States; Enable Medicine, Menlo Park, CA 94025, United States; Enable Medicine, Menlo Park, CA 94025, United States; Computational Biology Department, Carnegie Mellon University, Pittsburgh, PA 15213, United States; Enable Medicine, Menlo Park, CA 94025, United States; Enable Medicine, Menlo Park, CA 94025, United States; Department of Otolaryngology, University of Pittsburgh, Pittsburgh, PA 15213, United States; Enable Medicine, Menlo Park, CA 94025, United States; Computational Biology Department, Carnegie Mellon University, Pittsburgh, PA 15213, United States; Machine Learning Department, Carnegie Mellon University, Pittsburgh, PA 15213, United States

## Abstract

**Motivation:**

Spatial proteomics data have been used to map cell states and improve our understanding of tissue organization. More recently, these methods have been extended to study the impact of such organization on disease progression and patient survival. However, to date, the majority of supervised learning methods utilizing these data types did not take full advantage of the spatial information, impacting their performance and utilization.

**Results:**

Taking inspiration from ecology and epidemiology, we developed novel spatial feature extraction methods for use with spatial proteomics data. We used these features to learn prediction models for cancer patient survival. As we show, using the spatial features led to consistent improvement over prior methods that used the spatial proteomics data for the same task. In addition, feature importance analysis revealed new insights about the cell interactions that contribute to patient survival.

**Availability and implementation:**

The code for this work can be found at gitlab.com/enable-medicine-public/spatsurv.

## 1 Introduction

Recent technological advances have made it possible to study the expression patterns of many proteins simultaneously in a single *in situ* sample (Goltsev et al. 2018; Merritt et al. 2019; Hickey et al. 2022). These modalities, often termed “spatial proteomics” methods, have been used to map cell states to subcellular protein organization (Gut et al. 2018), build comprehensive single-cell maps of tissues (HuBMAP Consortium 2019), and further understand the tumor microenvironment (TME) in several cancers (Lewis et al. 2021), among several other applications (HuBMAP Consortium 2019; Hickey et al. 2022). Spatial proteomics imaging modalities (Goltsev et al. 2018; Merritt et al. 2019; Hickey et al. 2022) open the door to a more detailed characterization of spatial patterns of cells in tissue samples and their impact on patient outcomes. Another potential use of spatial proteomics is in clinical decision making. Recent advances in the analysis of cancer samples indicates that prognosis is linked to spatial pattern of immune and cancer cells and their interactions (Lewis et al. 2021). While such information can be obtained from spatial proteomics profiling, to date little work has focused on using such data for prediction and classification. A major challenge is to define the relevant spatial features that can be used as input to a prediction method.

The use of spatial data in prediction and forecasting tasks is well established in the fields of ecology and geology (Perry et al. 2006; Dowd et al. 2007; Velázquez et al. 2016). Spatial point processes (Baddeley et al. 2015) are often used in such fields to describe the spatial correlation between points, e.g. to describe the level to which points show aggregation/clustering or repulsion/inhibition. Such information is useful for tasks including testing for historical changes in spatial patterns of tree species (Sterner et al. 1986), estimating tree characteristics based on spatial patterns (Stoyan and Penttinen 2000), and predicting the occurrence of ore deposits for mining (Foxall and Baddeley 2002). In epidemiology, spatial information is often used for the prediction of patient outcomes and disease phenotypes (Pfeiffer et al. 2008); however, in such cases, the focus is on individuals and not interactions at the cellular level. While there have been some recent methods that take advantage of spatial proteomics data to improve the prediction of patient outcomes (Uttam et al. 2020; Wu et al. 2022b), to the best of our knowledge, a comprehensive analysis of spatial feature usage is still lacking for spatial proteomics data.

Here, we develop and use spatial features for supervised learning from spatial proteomics data. Specifically, we adapt spatial point process theory (Baddeley et al. 2015) for use in such data and use it to extract information on the spatial distribution of protein markers. We also define and use cell-based spatial features. These features are then used as input for survival prediction models for cancer prognosis analysis.

We applied our framework to analyze profiled head and neck squamous cell carcinoma (HNSCC) tumor samples from 81 patients. Roughly 600 000 individuals are diagnosed with HNSCC on an annual basis worldwide, with a 40%–50% mortality rate (Leemans et al. 2018). While in recent years there have been significant process in our understanding of the disease (Leemans et al. 2018; Cillo et al. 2020), there is still little understanding of the spatially relevant factors in the TME that contribute to the progression of the disease (Kulasinghe et al. 2021). As we show, by elucidating the spatial factors that affect clinical outcomes, our method can both improve diagnosis and treatment decisions and lead to better understanding of the molecular mechanisms impacting disease progression.

## 2 Methods

**Figure 1.**
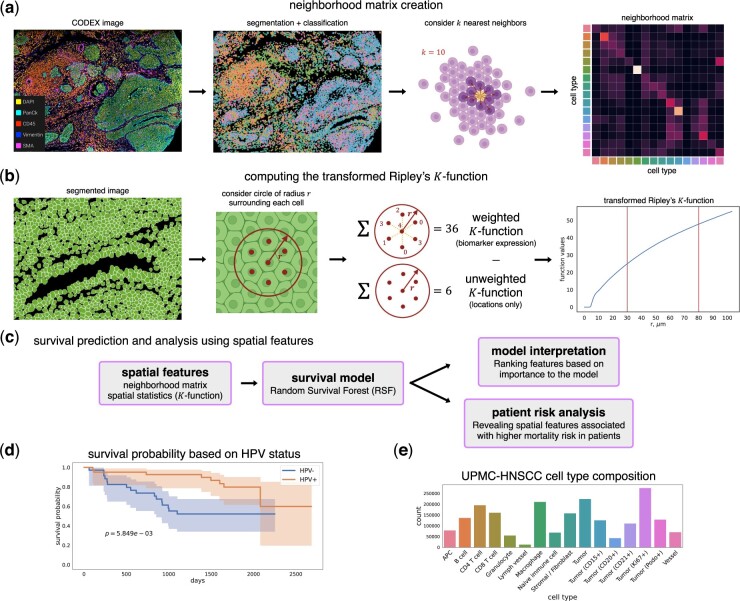
(a) Processing of data and creation of the neighborhood matrix. We first perform segmentation on the images and assign cell types based on biomarker expression. The neighborhood matrix is created by considering *k* = 10 nearest neighbors of each cell (Section 2). (b) The transformed version of Ripley’s *K*-function (Section 2) is used to extract biomarker-based spatial features from the image samples, with function values at *r* = 12, 30 μm corresponding to 1 and 2–3 cell distances. (c) The extracted spatial features are evaluated with a survival prediction model. Analysis of the predicted risk scores and the top features used by the model reveal spatial features relevant to HNSCC. (d) Kaplan–Meier plot showing survival probability of patients, separated by HPV status. Log-rank *P*-value (p=5.849e−3) indicates the difference between HPV− and HPV+ survival curves is statistically significant. (e) Cell type composition within the UPMC dataset. The colors match the colors in the second image of (a).

We introduce a framework to extract spatially relevant features from spatial proteomics imaging data for use in prediction of patient survival (Fig. 1). We then train a random survival forest (RSF) model (Ishwaran et al. 2008) using these features to predict low- and high-risk individuals (Fig. 4a) and compare the results to methods that use traditional, non-spatial, features.

### 2.1 Dataset description and pre-processing

The dataset used in this work is a 39-plex spatial proteomics CODEX dataset from 81 HNSCC patients. Details of the dataset collection can be found in Wu et al. (2022b) (Dataset UPMC-HNC). After performing quality control, which included removing samples with poor quality (few cells present, out of focus, etc.) or with poor biomarker staining performance (low signal-to-noise ratio, poor subcellular specificity, etc.), our dataset included 307 imaged samples collected from 7 batches/coverslips, with between 1 and 8 samples belonging to each patient. In this work, we excluded samples from “normal mucosa” tissue as we were only interested in using samples from cancerous tissue, resulting in a total of 281 samples and 1 973 232 cells, with an average of 7022 cells per sample. Samples were annotated with clinical data, including patient status, patient survival length, and Human papillomavirus (HPV) status (Supplementary Table S1 and Supplementary Fig. S1).

#### 2.1.1 Data split and evaluation

For performance evaluation, cross-validation sets were prepared in two ways:

1. *N*-fold cross validation, grouped by patients. *N* = 10 for the survival prediction task.
2. Leave-one-coverslip-out. For each fold, all the samples from a single coverslip are held out as the validation set, resulting in seven total folds.

Training and evaluation were run independently for each of the folds, and prediction performances were averaged over all folds.

### 2.2 Cell segmentation and classification

After image preprocessing, we applied a neural network-based cell segmentation tool, DeepCell (Greenwald et al. 2022), on the 4′,6-diamidino-2-phenylindole (DAPI) image channels to identify nuclei, and these nuclear masks were dilated to obtain whole-cell segmented cells. Nuclear segmentation masks were stochastically dilated by flipping pixels with a probability equal to the fraction of positive neighboring pixels. This dilation was repeated for nine cycles for all CODEX data (Wu et al. 2022a, 2022b). On each CODEX sample, given the segmentation of individual cells, single cell expression was computed for each biomarker *j* (Supplementary Methods) (Hickey et al. 2021; Wu et al. 2022a).

To assign cell types, we obtained an cell-by-marker expression matrix with normalized expression values $z(x_{i}^{\left(j\right)})$. Then we performed principal component (PC) analysis to extract the top 20 PCs. We constructed a *k*-nearest neighbor graph (*k* = 30) on top of the 20 PCs of the expression matrix and performed graph clustering (Traag et al. 2019) on the result. Clusters were manually annotated according to their cell biomarker expression patterns (Supplementary Fig. S2). This procedure was performed on a subset of 10 000 cells and subsequently used to train a *k*-nearest neighbor algorithm to predict cell types from the normalized expression vector. This algorithm was used to transfer labels to the entire dataset.

### 2.3 Non-spatially dependent features

We extracted several non-spatial features from the data.

**Average biomarker across image**: For each sample, a 39-length vector is computed with the average biomarker pixel intensity across the sample image for each biomarker.

**Average biomarker in cells**: For each sample, a 39-length vector is computed with the average biomarker pixel intensity within the cell segmentation masks for each biomarker.

**Cell type proportions**: For each sample, a 16-length vector is computed with the fraction of each cell type present in the sample. This vector sums to 1.

### 2.4 Spatially dependent features

To take advantage of the spatial information present in the data, we developed several spatial-based features as follows:

#### 2.4.1 Neighborhood matrix

For each sample, we compute a “neighborhood matrix,” *M*, which is a matrix with shape (number of cell types) × (number of cell types), and each row sums to 1. Here, *M* is a 16 × 16 matrix. Element *m_ij_* is the fraction of cell type *j* within the *k*-nearest neighbors of cell type *i*, averaged across all cells of type *i* in the sample. In this work, we use *k* = 10, and nearest neighbors are computed using the centroid coordinates of each cell. We observe similar results for other values of *k*.

#### 2.4.2 Spatial statistics

We leveraged techniques based on spatial point process theory, which is widely used in the fields of ecology and geology (Perry et al. 2006; Dowd et al. 2007; Baddeley et al. 2015; Velázquez et al. 2016). A spatial point pattern is a dataset that gives the spatial locations of things or events. A point process is a spatial probability distribution whose outcome is a spatial point pattern. A common null model for a point process is the “homogeneous” “Poisson point process,” which exhibits “complete spatial randomness” (CSR). This process is characterized by two key properties: (i) homogeneity: the points have no preference for any spatial location and (ii) independence: information about the outcome in one region of space has no influence on the outcome in other regions. A spatial point pattern diverging from this null model indicates spatial clustering or dispersion. This information is used as a feature in our analysis for spatial point patterns of specific biomarkers.

#### 2.4.3 A transformation of Ripley’s *K*-function

A popular technique for analyzing spatial correlation and measuring deviation from CSR in point patterns is Ripley’s *K*-function (Baddeley et al. 2015). Informally, this function is a measure of the pairwise distance distribution of points in the pattern and can indicate whether points are clustered, dispersed, or distributed randomly. The *K*-function of a stationary point process *X* is defined such that λK(r) is equal to the expected number of additional random points within a distance *r* of a typical random point of *X*. Here, *λ* is the intensity of the process, i.e. the expected number of points of *X* per unit area. The *K*-function can be estimated using where *A* is the area of the window, *n* is the number of data points, *d_ij_* is the distance between points *i* and *j* in *X*, and $1(d_{ij}\leq r)$ is an indicator that equals 1 if the distance is less than or equal to *r*. $e_{ij}(r)$ is an edge correction weight. Specifically, we use Ripley’s isotropic correction estimator (Supplementary Methods).


(1) $$\hat{K}(r)=\frac{A}{n(n-1)}\sum_{i=1}^{n}\sum_{i=1,j\neq i}^{n}1(d_{ij}\leq r)e_{ij},$$

The *K*-function for a homogeneous Poisson point process is $K_{\text{pois}}=\pi r^{2}$ (Baddeley et al. 2015). One commonly used transform of the *K*-function is Besag’s *L*-function (Baddeley et al. 2015), which can be estimated with $\hat{L}(r)=\sqrt{\frac{\hat{K}(r)}{\pi }}$. This transforms the theoretical Poisson *K*-function to the straight line $L_{\text{pois}}(r)=r$, which makes visual assessment of the graph easier during exploratory analysis.

Spatial point processes and patterns can be “marked,” which means that each point is associated with a discrete or continuous value. For example, if a cell is represented as a point, its mark can be the vector of biomarker expression values for that cell. The mark-weighted *K*-function for marked point processes is a generalization of Ripley’s *K*-function, in which the contribution from each pair of points is weighted by a function of their marks. This function allows us to measure the spatial correlation of mark values in addition to the spatial correlation of the points themselves. We estimate the mark-weighted *K*-function for each cell (Supplementary Methods), denoted as ${\hat{L}}_{w,m}(r)$ for feature *m*.

#### 2.4.4 Using spatial statistics to derive spatial features

We use the above spatial statistics techniques to featurize our data as follows:

1. We assume that each cell centroid represents a point in a spatial point process and that the normalized biomarker expression values for that cell are the “marks” or weights for that point.
2. Compute $\hat{L}(r)$ using cell centroid coordinates.
3. For each biomarker *m*, compute ${\hat{L}}_{w,m}(r)$, the *L*-transformation of the mark-weighted *K*-function.
4. Compute ${\hat{L}}_{w,m}^{\text{norm}}(r)={\hat{L}}_{w,m}(r)-\hat{L}(r)$ for each biomarker in the sample. By subtracting the unweighted *L* from the weighted version, we can inspect whether individual biomarker expressions are clustered or dispersed with respect to the cell locations.

$\hat{L}(r)$ and ${\hat{L}}_{w,m}(r)$ were computed using the Lest and Kmark functions from the R spatstat package (Baddeley and Turner 2005), respectively. For each sample, we computed ${\hat{L}}_{w,m}^{\text{norm}}(r)$ for r∈{0,1,…,400} pixels for each biomarker *m*. We treated *r* as a hyperparameter, which determined which values of ${\hat{L}}_{w,m}^{\text{norm}}(r)$ to use as input, and evaluated the performance of our prediction models using different values of *r* (Supplementary Figs S3 and S4). From these experiments, we chose the values r∈{80,212}, which correspond to around 30 and 80 μm, the approximate diameter of 2–3 and 6–8 cells, respectively. This resulted in a (39*2)-length vector for each sample as input to our models.

### 2.5 Comparison methods

We compared the use of our spatial features with several prior methods (Patwa et al. 2021; Seal et al. 2022). While these were applied to spatial data for clinical outcome prediction, unlike our approach, they do not fully utilize spatial features.

#### 2.5.1 Biomarker positivity and interactions based on thresholds

A commonly used method for characterizing cells from fluorescence imaging data is to convert biomarker expression in each cell to a binary indicator using a threshold (Patwa et al. 2021). Following the methods of Patwa et al. (2021), we calculated the biomarker positivity thresholds from expression levels of the image background. The image background is all pixels in the sample that are not assigned to cells after cell segmentation. We would expect that the pixel intensities outside of cells are close to zero, and so an intuitive way to choose a threshold is to take the average pixel values in the image background. For each biomarker, we summed the intensity values in the image background across all samples and divided this value by the total number of background pixels to get the average background intensity. This value was used as the threshold to determine whether a cell was positive for that biomarker. For each sample, we then computed the fraction of cells positive for each biomarker using the thresholds, resulting in a 39-length vector for each sample as input to our models.

We also computed biomarker interactions between neighboring cells, again following the methods from Patwa et al. (2021). See Supplementary Methods for details.

#### 2.5.2 DenVar

DenVar (Seal et al. 2022) is an approach to cluster spatial proteomic imaging samples/subjects into meaningful groups based on the probability densities of biomarker expression. For each sample, the probability density of each biomarker is estimated using kernel density estimation. Next, the Jenson–Shannon distance (Endres and Schindelin 2003) is used to quantify the difference between densities of the same biomarker in pairs of samples. To classify samples using this approach, we used the matrix of distances between samples to separate the samples into two clusters using hierarchical clustering. These cluster assignments were computed for each biomarker, resulting in a 39-length binary vector for each sample indicating the cluster assignment for each biomarker.

### 2.6 Survival analysis

#### 2.6.1 Right-censored survival data

In right-censored survival data, the observed data for each sample is (T,δ), where *T* is the observed time and δ∈{0,1} is an event/censorship indicator, where 0 means the observation is censored (patient is alive) and 1 means an event has occurred (patient has died). When *δ* = 1, we know that *T* is the true survival time, but when *δ* = 0, we only know the patient has survived to time *T*, but not when they actually die.

#### 2.6.2 RSF

RSFs (Ishwaran et al. 2008) are an extension of random forests to the setting of right-censored survival data. We refer to the individual trees within an RSF as survival trees. Here we performed RSF analysis for the cancer data. See Supplementary Methods for details on the RSF implementation.

For the evaluation of the RSF models, we use Harrell’s *C* index, or concordance index, for right-censored data. See Supplementary Methods for details.

### 2.7 Stratifying patients into “low”- and “high”-risk cohorts

To stratify patients into “low-risk” and “high-risk” cohorts, we used the predicted risk values from the RSF model trained on the neighborhood matrix. The model output a risk score for each sample, and we averaged these scores across samples for each patient to obtain a patient-level risk score. Next, we chose a threshold that maximized the difference between the Kaplan–Meier curves for the resulting cohorts based on the log-rank test (Peto and Peto 1972). We used the Mann–Whitney *U*-test with a Bonferroni multiple hypothesis test correction to determine statistically significant differences in neighbor fraction values between the two cohorts.

## 3 Results

We developed spatial-based feature extraction methods for spatial proteomics data. These include a matrix encoding neighborhood relationships between pairs of cell types and a spatial point process method that measures spatial correlation, a transformed version of Ripley’s *K*-function (Fig. 1). We used these spatial features to predict survival times of HNSCC patients and assign “low”- and “high”-risk patient cohorts based on the spatial and protein marker data. To evaluate our features, we used a 39-plex spatial proteomics CODEX dataset from 81 HNSCC patients (Section 2).

### 3.1 Data featurization

Table 1 shows different properties of each of the extracted features from the data. Note that these features can be largely divided based on their use (or lack thereof) of spatial information. For example, “average biomarker across image” and “average biomarker in cells” do not utilize specific location and can be computed by non-spatial methods. In contrast, the “neighborhood matrix” can only be computed if spatial data are available. We denote each feature type as spatially or non-spatially dependent in Table 1.

**Table 1.** Different featurizations of the data.

Featurization	Spatially dependent	Cell segmentation	Cell type-based	Biomarker-based	Pairwise
Average biomarker across image	✗	✗	✗	✓	✗
Average biomarker in cells	✗	✓	✗	✓	✗
Cell type proportions	✗	✓	✓	✗	✗
Neighborhood matrix	✓	✓	✓	✗	✓
Ripley’s *K* function, ${\hat{L}}_{w,m}^{\text{norm}}(r)$	✓	✓	✗	✓	✗

“Spatially dependent” means that the featurization uses the spatial locations of cells, “cell segmentation” uses the cell segmentation masks, “cell type-based” uses the cell type labels, “biomarker-based” uses the biomarker expression levels “directly” in the featurization, and “pairwise” computes pairwise features between cell types.

### 3.2 Ripley’s *K*-function features describe clustering and dispersion patterns in spatial proteomics data

**Figure 2.**
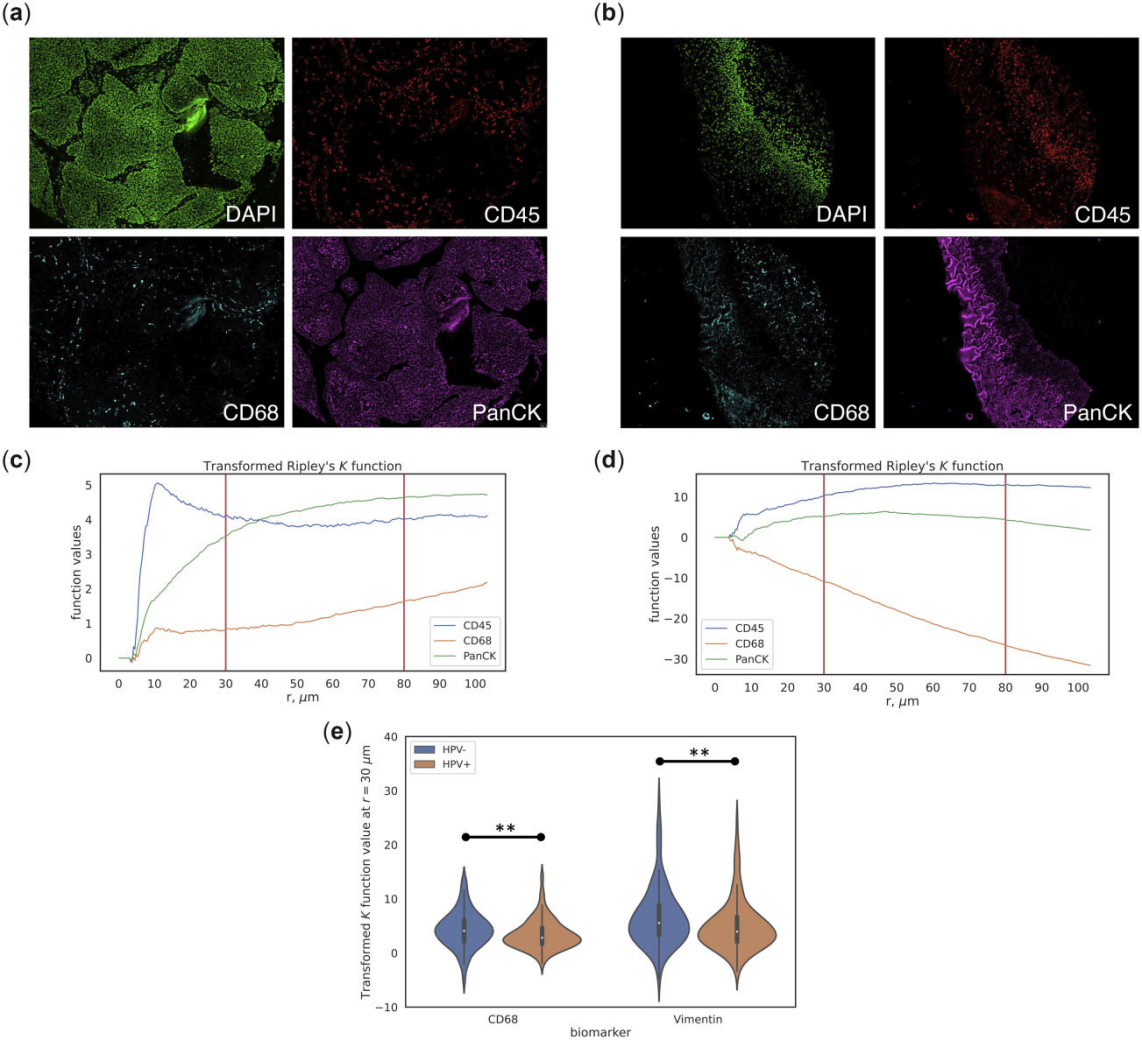
(a, b) Image samples showing biomarker intensity of DAPI, CD45, CD68, and PanCK. (c, d) Transformed Ripley’s *K*-function for markers CD45, CD68, and PanCK corresponding to (a) and (b), respectively. (e) Violin plot of transformed *K* function values at *r* = 30 μm for biomarkers CD68 and Vimentin, separated by HPV status. ** indicates Bonferroni-corrected *P*-value < .05.

Ripley’s *K*-function (Baddeley et al. 2015) is a measure of spatial correlation between points in a point pattern and can indicate whether points are clustered or dispersed. We computed a novel transformed version of the *K*-function to inspect clustering and dispersion behavior of biomarker expression with respect to cell locations (Section 2). Figure 2a–d shows some examples of this function applied to the data. In general, lower function values correspond to more dispersed biomarker expression (CD68 in both examples), and higher function values correspond to clustered expression at that value of *r* (CD45 in both examples).

In HNSCC, HPV status is a key prognostic factor; patients that test positive for HPV usually have a better survival rate than patients without HPV (Leemans et al. 2018), which is reflected in this dataset (Fig. 1e). In order to reveal spatial patterns that differ between HPV− and HPV+ patients, we used the transformed *K*-function values at *r* = 30, 80 μm, which approximate the diameter of 2–3 and 6–8 cells, respectively, to inspect the difference in function values between these cohorts. We found several biomarkers that exhibited significantly different values between these cohorts (Supplementary Table S2). For example, at *r* = 30 μm (small cell clusters), CD68 and Vimentin function values in the HPV− cohort are significantly greater than those in the HPV+ cohort (Fig. 2e, *P* < .05), indicating increased clustering behavior of these biomarker expressions in the HPV− samples. It has been shown that increased density of CD68+ tumor-associated macrophages (TAMs) is associated with increased vascular and lymphatic invasion in HNSCC (Pollard 2004; Kuang et al. 2007), and Vimentin is known to be associated with tumor growth and metastasis in cancer (Satelli and Li 2011). These differences in the transformed *K*-function values suggest that increased spatial clustering of these biomarkers may be correlated with the worse prognosis often found in HPV− patients of HNSCC.

### 3.3 Spatially dependent feature sets improve predictive performance

**Figure 3.**
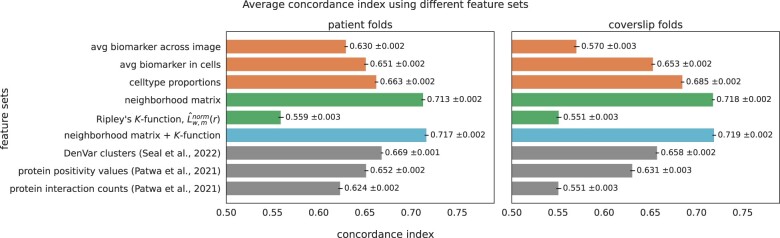
Survival length prediction performance using different feature sets. The colors of the bars mean the following; orange: non-spatial features, green: spatial features, blue: combination of spatial features, gray: comparison methods. Concordance indices are presented with 95% confidence intervals. The combination of the neighborhood matrix and the transformed Ripley’s *K*-function has the highest average concordance index for both the patient-grouped and coverslip-grouped cross-validation schemes.

To highlight the usefulness of spatial information for prediction, we compared spatially dependent and non-spatially dependent features by training a RSF model to predict survival lengths in HNSCC patients. For this analysis, we used two cross-validation schemes: (i) 10-fold cross-validation where folds were grouped by patients and (ii) 7-fold cross-validation where each fold is a different coverslip/batch (Section 2). We computed the average concordance index on the held-out test set for each fold, and repeated each experiment 100 times with different random seeds. We found that using the spatially dependent neighborhood matrix leads to higher performance in the survival prediction task (Fig. 3, 7.5% and 4.8% increase compared with the top-performing non-spatial features for the patient and coverslip folds, respectively). In addition to the neighborhood matrix, we also tested the usage of the transformed Ripley’s *K*-function for survival prediction. While using it on its own does not improve upon the other individual features, when combined with other spatial features (the neighborhood matrix) it obtains the highest average concordance index (0.717 and 0.719 for patient and coverslip folds, respectively).

We performed additional experiments combining spatial and non-spatial features to train RSF models (Supplementary Fig. S5). We found that while combining the neighborhood matrix with the non-spatial features improved performance over using the non-spatial features alone (9.2% and 13.4% for patient and coverslip folds, respectively), none of these combinations improve performance over the combination with the neighborhood matrix and the *K*-function features.

We also compared the performance of the spatial features to other methods that have been previously applied to classifying proteomic imaging data. Specifically, we compared our features with DenVar (Section 2), an approach to cluster spatial proteomic imaging samples into meaningful groups based on probability densities of biomarker expression (Seal et al. 2022), and to an approach by Patwa et al. (2021), which defines thresholds to convert biomarker expressions in each cell to a binary value. The proportion of cells positive for each biomarker in a sample is then used as input for the RSF model (Section 2). Results for these methods, presented in the gray bars in Fig. 3, show that combining the different spatial features we propose improves upon these previous methods (7.2% and 9.3% increase compared with the top-performing comparison method for the patient and coverslip folds, respectively).

### 3.4 Top features selected by the RSF model are relevant to HNSCC

We inspected the top features utilized by the RSF model by using the permutation importance method (Altmann et al. 2010) to rank features. In this method, each feature is randomly permuted across samples *n* = 100 times and the average change in concordance index is computed. The larger the change in concordance index, the higher the feature ranks in importance. Supplementary Table S3 shows the top 15 features for each of the feature sets listed in Fig. 3. Many of the top features are known to be associated with survival. For example, the top-ranked feature from the RSF trained using “cell type proportions” features is a CD21-positive tumor cell subtype. While the mechanisms of this specific tumor subtype in HNSCC are still under-explored, the proportion of tumor cells in a sample is associated with worse prognosis in some cancers (West et al. 2010). Furthermore, most of the top-ranked spatial features from the “neighborhood matrix” involve pairwise interactions between different tumor cell subtypes. These features can be interpreted as measures of tumor heterogeneity and suggest that such heterogeneity plays a key role in disease progression. Indeed, previous work has shown that tumor heterogeneity correlates with poor outcome in HNSCC (Mroz and Rocco 2013). From the RSF model trained on both the neighborhood matrix and *K*-function features, the top *K*-function feature was the FoxP3 function value at *r* = 80 μm. FoxP3 is a marker for regulatory T cells, which has been shown to upregulate immunosuppressive molecules in HNSCC tumors (Jie et al. 2013).

### 3.5 RSF-derived risk factors reveal enriched cell–cell interactions in “high-risk” HNSCC patients

**Figure 4.**
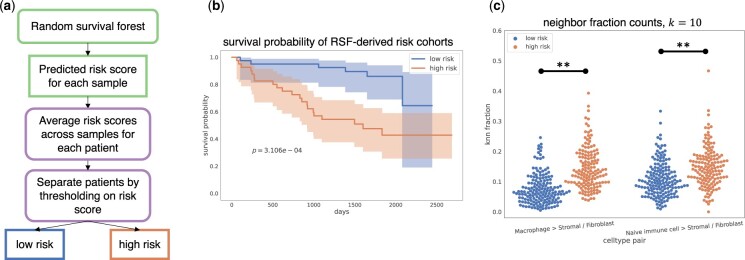
Immune cell-fibroblast interactions are enriched in tumors of “high-risk” HNSCC patients. (a) Predicted risk values from RSF are used to stratify patients into “low-risk” and “high-risk” cohorts. (b) Kaplan–Meier curves of “low-risk” and “high-risk” patients with log-rank *P*-value (p=3.106e−4). (c) Nearest neighbor fraction values for cell type pairs (Macrophage <> Stromal/Fibroblast) and (Naive immune cell <> Stromal/Fibroblast) in “low-risk” and “high-risk” samples. ** indicates Bonferroni-corrected *P*-value < .01.

We next used predicted risk values from the RSF model trained on the neighborhood matrix features to stratify patients into “low-risk” and “high-risk” cohorts (Fig. 4a; Section 2). Figure 4b shows that the Kaplan–Meier curves for these cohorts are statistically significantly different (log-rank test, p=3.106e–4). We performed statistical analysis on the neighborhood features (Section 2) and revealed that there were significant differences in the neighbor fraction counts for macrophage-stromal/fibroblast interactions (Mann–Whitney *U*-test, p=4.10e–19) and naive immune cell-stromal/fibroblast interactions (p=9.26e–7) between the “low-risk” and “high-risk” cohorts (Fig. 4c). An increase in macrophage-stromal/fibroblast interactions in high-risk patients may suggest that macrophages have been recruited to the tissue surrounding the tumor to suppress the immune response; however, this hypothesis would need to be explored further to be confirmed. TAMs are known to suppress antitumoral immunity and promote tumor progression (Pollard 2004; Kuang et al. 2007), and a high density of TAMs have been shown to be associated with poor prognosis in many cancers (Pollard 2004; Kuang et al. 2007). There is also evidence of cancer-associated fibroblasts (CAFs) inducing the immunosuppressive and protumoral phenotype of TAMs in oral squamous cell carcinoma (Takahashi et al. 2017). The increase in naive immune cell (CD45RA+)-stromal/fibroblast interactions in high-risk patients is supported by previous studies showing that CAFs are able to modulate tumor-associated T cell activity (Nazareth et al. 2007) and encourage immune cells to promote the development of tumors in many cancers (Mao et al. 2021). For example, such interactions can facilitate the degradation of the extracellular matrix, which results in dysfunction in the cancer immune response (Ziani et al. 2018; Mao et al. 2021). A full list of significant pairwise neighbor interactions can be found in Supplementary Fig. S6.

## 4 Discussion

Spatial proteomics is a promising direction for the study, analysis, and use of clinical tissue samples. While some work has already been applied to predict clinical outcomes using this data, few methods fully utilize the available spatial information. Motivated by prior work in ecology and epidemiology, we explored novel spatial features that can be extracted from spatial proteomics data. These included features based on cell type location (neighborhood matrix) and features based on individual markers (a transformation of Ripley’s *K*-function).

We tested the use of these new spatial features to predict the survival length of patients with HNSCC. Incorporation of spatial features into predictive models led to consistent performance improvements over both non-spatial features and previous methods that did not fully utilize spatial information. Specifically, the combination of Ripley’s *K*-function and neighborhood matrix features performed best. Interestingly, we observed that combining the *K*-function and non-spatial features do not improve much over only using non-spatial features (Supplementary Fig. S5). This may suggest that the neighborhood matrix complements the *K*-function for performance improvement, and exploration of how this occurs is an interesting direction for future work.

While the main goal of these features is to improve patient outcome prediction, they can also be used to explore interactions that impact survival. Predicting patients as low risk and high risk using our survival model revealed cell type interaction pairs from the neighborhood matrix that are significantly enriched in high-risk HNSCC patients, namely macrophage-stromal/fibroblast interactions and naive immune cell-stromal/fibroblast interactions. The model predicts that an increase in interactions between these pairs leads to higher risk patients. These results agree with previous findings that suggest that interactions between TAMs and CAFs lead to immunosuppressive and protumoral behavior which increases risk (Pollard 2004; Kuang et al. 2007; Mao et al. 2021).

Our work can also be used to motivate additional types of features. These include the use of unsupervised methods to cluster cells rather than supervised cell type assignment when constructing the neighborhood matrix and non-cell-based *K*-functions for biomarkers. While these methods were not explored in this article, they are a promising direction for future work.

Given its advantages, spatial proteomics data will continue to gain popularity in clinical sample analysis. We believe that the methods presented would enable better use of the data leading to improve biological inference and decision making.

## Supplementary Material

btad245_Supplementary_Data

## Data Availability

Cell expression data, cell coordinates, computed features, and data to reproduce cohort analyses are available in Zenodo, with the identifier https://doi.org/10.5281/zenodo.7796393. Raw image data are available from the corresponding author upon reasonable request.
