# Supplementary material for: Deriving spatial features from in situ proteomics imaging to enhance cancer survival analysis
Source: Bioinformatics. 2023 Jun 30;39(Suppl 1):i140–8. doi: 10.1093/bioinformatics/btad245 (PMC10311350; doi:10.1093/bioinformatics/btad245)
Supplement: btad245_Supplementary_Data [file btad245_supplementary_data.pdf]

Supplementary Material: Spatial feature extraction for enhancing cancer survival analysis in spatial proteomics imaging data

Monica T. Dayao[1,2], Alexandro Trevino[3], Honesty Kim[3], Matthew Ruffalo[2], H. Blaize D'Angio[3], Ryan Preska[3], Umamaheswar Duvvuri[4], Aaron T. Mayer[3], and Ziv Bar-Joseph[2,5]

[1]Joint Carnegie Mellon University-University of Pittsburgh Ph.D. Program in Computational Biology, Pittsburgh, PA
[2]Computational Biology Department, Carnegie Mellon University, Pittsburgh, PA
[3]Enable Medicine, Menlo Park, CA
[4]Department of Otolaryngology, University of Pittsburgh, Pittsburgh, PA
[5]Machine Learning Department, Carnegie Mellon University, Pittsburgh, PA

1 Supplementary Methods

1.1 Cell expression normalization

We perform the following steps to normalize cell biomarker expression.

1. Compute the mean expression value across pixels within the cell segmentation mask. Denote the mean expression value of cell i as $x_i^{(j)}$, and denote the array of all expression values $\{x_1^{(j)}, x_2^{(j)}, ...\}$ as $X^{(j)}$.

2. Normalize the expression value using quantile normalization and inverse sine transformation:

$$f(x_i^{(j)}) = \operatorname{arcsinh}\left(\frac{x_i^{(j)}}{5Q(0.2; X^{(j)})}\right) \tag{1}$$

   where $Q(0.2; X^{(j)})$ represents the 20th quantile of $X^{(j)}$, and arcsinh is the inverse hyperbolic sine function. We can denote the array of normalized expression values for biomarker j as $\{f(x_1^{(j)}), f(x_2^{(j)}), ...\}$ as $f(X^{(j)})$. We use these values for computing the transformed Ripley's K-function as described in the main Methods section.

3. Calculate the z-score of the normalized expression value:

$$z(x_i^{(j)}) = \frac{x_i^{(j)} - \mu}{\sigma} \tag{2}$$

   where μ and σ are the mean and standard deviation of $f(X^{(j)})$, respectively.

These normalized values are used to assign cell types (Supplementary Figure 2).

1.2 Ripley's isotropic correction estimator

$$e_{ij} = \frac{1}{p(x_i, d_{ij})} \tag{3}$$

where $p(x_i, d_{ij})$ is the fraction of the length of a circle centered at x_i with radius d_{ij} that lies within the window. This correction method treats edge effects as a form of sampling bias and assumes that the point process is isotropic (statistically invariant under rotation).

1.3 Estimation of the mark-weighted K-function

The mark-weighted K-function [1] can be estimated using

$$\hat{K}_{w,m}(r) = \frac{A}{(\sum_i^n m_i)^2} \sum_{i=1}^{n} \sum_{i=1, j \neq i}^{n} m_i m_j \mathbf{1}(d_{ij} \leq r) e_{ij} \tag{4}$$

where m_i and m_j are the mark values for feature m for points i and j respectively. Similar to the K-function, the mark-weighted K-function can be transformed to

$$\hat{L}_{w,m}(r) = \sqrt{\frac{\hat{K}_{w,m}(r)}{\pi}} \tag{5}$$

1.4 Computing Patwa et al. biomarker interactions [2]

We created Voronoi tessellations using the cell centroids, with adjacent Voronoi regions representing interactions between cells. Given two interacting cells, we constructed two lists: List 1 contained the names of the proteins that Cell 1 was positive for, and List 2 contained the names of the proteins that Cell 2 was positive for, using the thresholds as defined previously. We took the Cartesian product of the two lists to find all of the combinations of proteins present in this interaction. For each sample, we tallied all the biomarker interactions between interacting cells into a biomarker-biomarker matrix. We took the top half of the symmetric matrix and flattened it to create a 780-length feature vector for each sample.

1.5 Random survival forest (RSF) details

There are two key differences between traditional random forests and RSFs: 1) the splitting rule at each node within a tree, and 2) the predicted value for each sample. To handle event censoring times, RSFs employ a splitting rule that maximizes the log-rank test statistic at each node [3]. The log-rank test statistic is traditionally used for two-sample testing of survival distributions. Instead of predicting the length of survival for a patient as one may expect with a traditional random forest for regression, a RSF predicts the *ensemble mortality* for each sample, which measures the expected number of deaths/events at the given time under a null hypothesis of similar survival behavior.

We can define $d_{l,h}$ and $Y_{l,h}$ to be the number of deaths and individuals, respectively, at time $T_{l,h}$. h is a unique terminal node in the survival tree, and l denotes the distinct event times in the data. $\mathbf{x}_i$ is the d-dimensional covariate for sample i. Due to the binary nature of survival trees, each $\mathbf{x}_i$ falls into a unique terminal node h. In each survival tree in the forest, the cumulative hazard function (CHF) for sample i can be estimated with

$$H(T|\mathbf{x}_i) = \sum_{T_{l,h} \leq T} \frac{d_{l,h}}{Y_{l,h}} \quad \text{if } \mathbf{x}_i \in h \tag{6}$$

The ensemble CHF, $H_e(T|\mathbf{x}_i)$ can be estimated by taking the average of $H(T|\mathbf{x}_i)$ across all survival trees in the forest. The ensemble mortality, or ensemble risk score, can then be computed with

$$M_i = \sum_{j=1}^{n} H_e(T_j|\mathbf{x}_i) \tag{7}$$

where n denotes the total number of distinct death times in the training data. In this work, we used `RandomSurvivalForest` from the Python package `scikit-survival` for the RSF implementation [4]. For predicting risk scores for each sample, we used the `RandomSurvivalForest.predict` function.

1.6 Concordance index

The concordance index $\hat{c}$ [5] is computed as follows, where C is the number of concordant pairs, D is the number of discordant pairs, and R is the number of pairs with the same risk value. Pairs are only comparable if both patients experience an event (not censored), or the patient with the shorter survival time experienced an event. Pairs that do not

satisfy either of these criteria are not considered in the concordance index.

$$\hat{c} = \frac{C + R/2}{C + D + R} \tag{8}$$

$\hat{c} = 1$ means that all comparable pairs are concordant, and $\hat{c} = 0$ means that all comparable pairs are discordant. We used the `RandomSurvivalForest.score` method from the Python package `scikit-survival` to compute $\hat{c}$ [4].

2 Supplementary Tables and Figures

		samples (all)	samples (excl normal)	patients
patient status	NED	197	181	52
	DOD	57	54	16
	DOC	46	41	11
	AWD	7	5	2
HPV status	HPV-	128	112	35
	HPV+	158	148	41
	unknown	21	21	5
tissue type	primary tissue	183	183	–
	nodal metastasis	98	98	–
	normal mucosa	26	0	–
N_{samples}		307	281	
N_{patients}		81	81	
N_{batch}		7	7	
N_{cells}		2057701	1973232	

Table 1: HNSCC dataset details and relevant clinical annotations. The 'samples (excl normal)' column excludes all 'normal mucosa' samples. For patient status, 'NED' = 'no evidence of disease', 'DOD' = 'died of disease', 'DOC' = 'died of complications', and 'AWD' = 'alive with disease'.

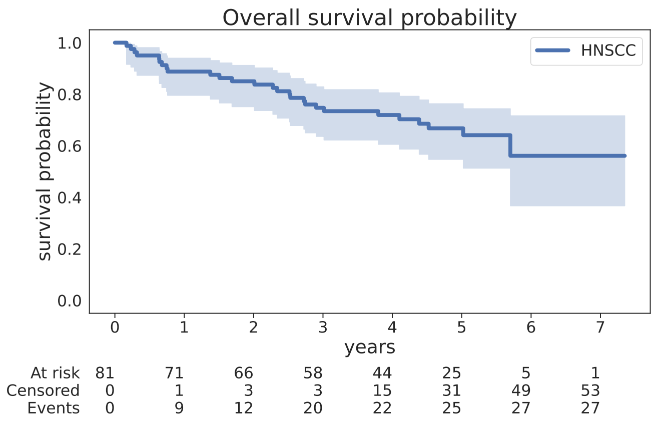

Figure 1: Kaplan-Meier curve showing survival probability for the HNSCC dataset ($n = 81$ patients).

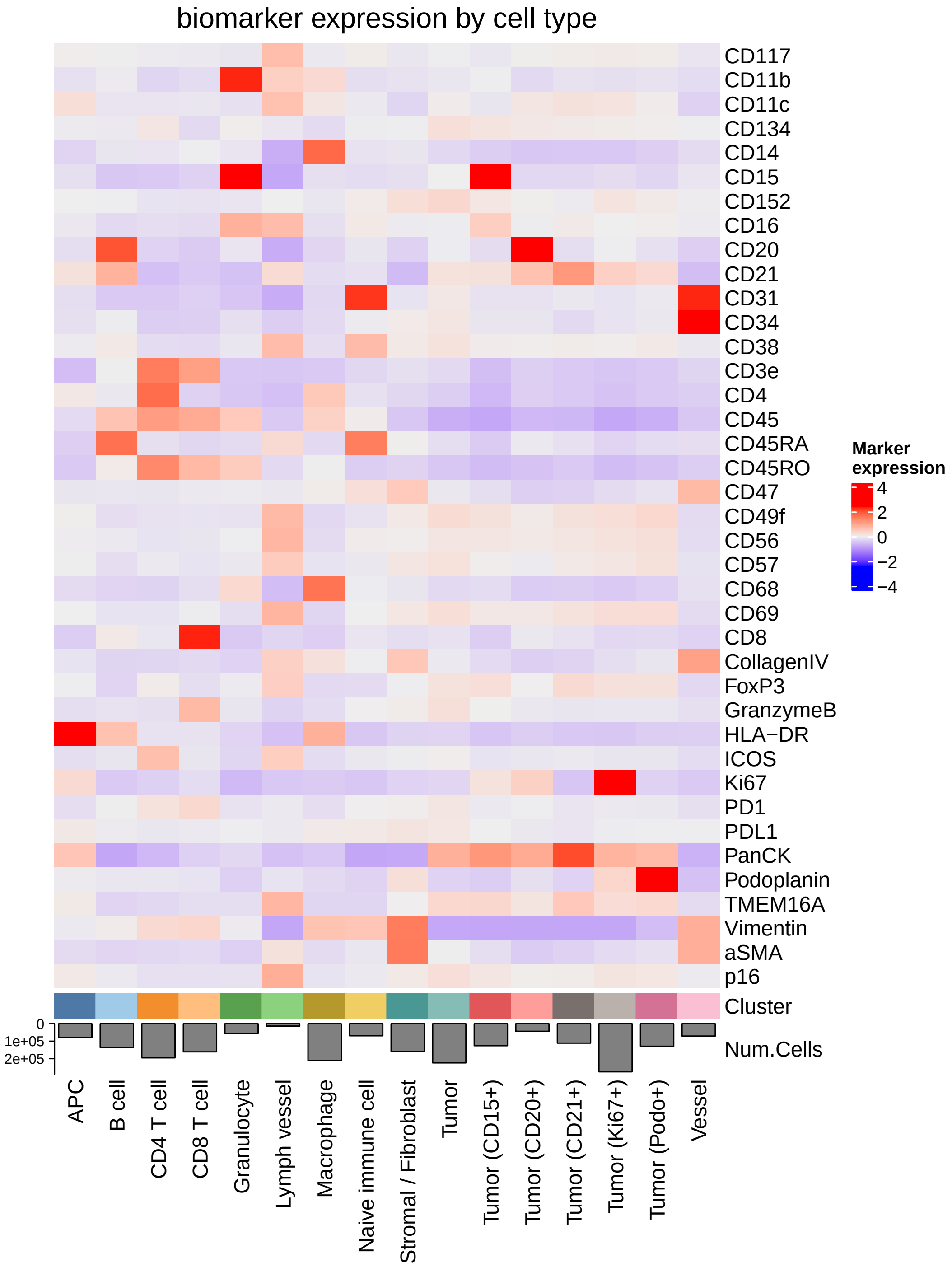

Figure 2: Normalized biomarker expression heatmap by cell type. Bar plot on the bottom axis indicates the cell type counts across the dataset.

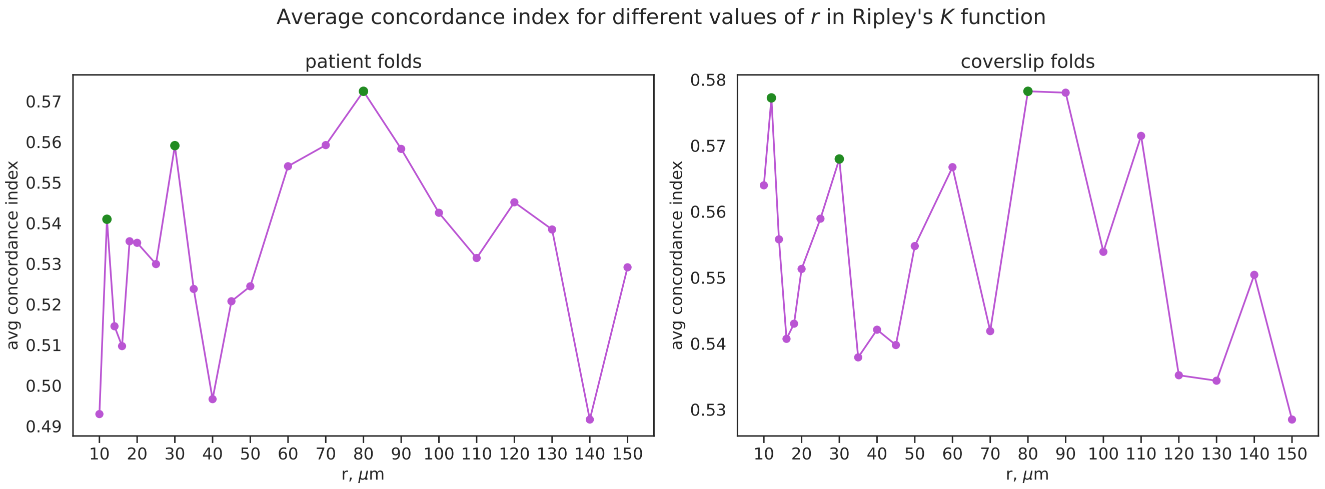

Figure 3: Average concordance index across cross-validation folds for random survival forest models trained on Ripley's K function features at different values of r. r values marked in green (12, 30, 80 μm) were selected for further experiments.

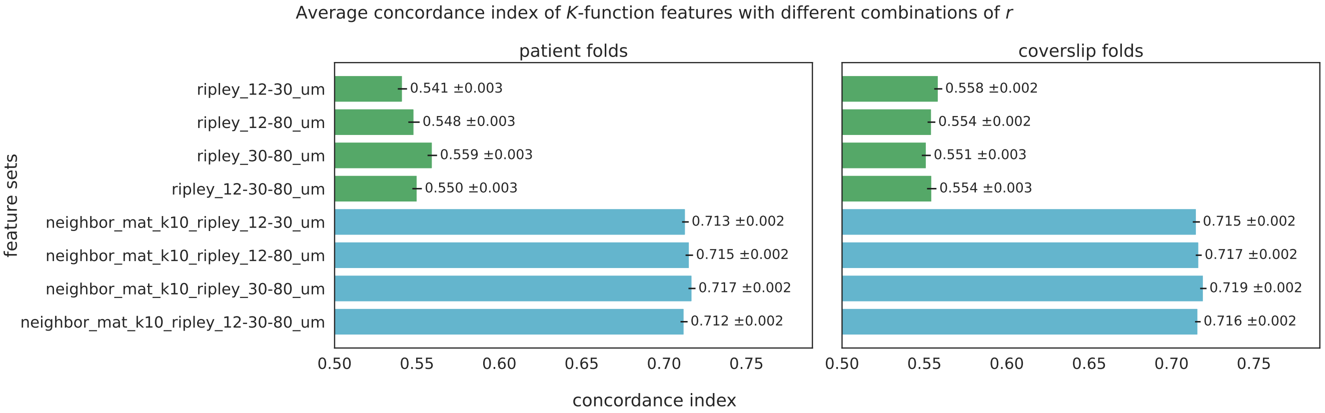

Figure 4: Average concordance index for random survival forest models trained using 100 different random seeds, with Ripley's K function features at different combinations of r ($r = 12, 30$ μm, $r = 30, 80$ μm, $r = 12, 30, 80$ μm). Concordance indices are presented with 95% confidence intervals. Green: using Ripley's K-function features only, blue: combining K-function with neighborhood matrix features.

$r = 12$ μm			$r = 30$ μm			$r = 80$ μm		
biomarker	$</>$?	corrected p-value	biomarker	$</>$?	corrected p-value	biomarker	$</>$?	corrected p-value
CD20	$<$	1.768e-02	CD68	$>$	4.398e-02	CD20	$<$	4.817e-02
CD45	$>$	9.543e-03	CollagenIV	$>$	1.374e-03	CollagenIV	$>$	1.342e-02
CD45RA	$<$	3.174e-02	Vimentin	$>$	4.192e-02			
CollagenIV	$>$	5.896e-03						
Vimentin	$>$	4.817e-02						

Table 2: Biomarkers that exhibited significantly transformed K-function values (at $r = 12, 30, 80$ microns) between HPV- and HPV+ sample cohorts. $<$ indicates that the function values were lower in the HPV- cohort than in the HPV+ cohort, $>$ vice versa. p-value was computed using a Mann-Whitney U-test with a Bonferroni multiple hypothesis test correction.

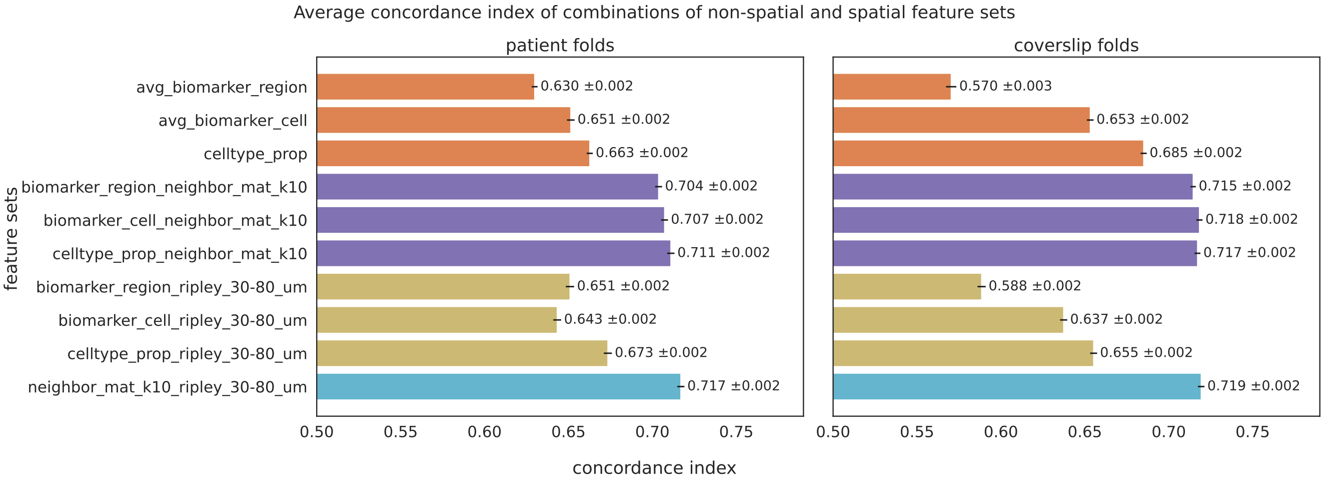

Figure 5: Average concordance index for random survival forest models trained using 100 different random seeds, with combinations of non-spatial and spatial feature sets. Concordance indices are presented with 95% confidence intervals. Orange: using non-spatial features, purple: combining non-spatial features with neighborhood matrix features, gold: combining non-spatial features with K-function features at $r = 30, 80$ μm, blue: combining neighborhood matrix and K-function features at $r = 30, 80$ μm.

rank	avg biomarker across image	avg biomarker in cells	celltype proportions	neighborhood matrix	neighborhood matrix + K-function
1	CD8	aSMA	Tumor (CD21+)	Tumor (Podo+) > Tumor(CD21+)	Tumor (Ki67+) > B cell
2	CD20	HLA-DR	APC	Tumor (Ki67+) > B cell	Tumor (Podo+) > Tumor (CD21+)
3	CD45	CD45R0	B Cell	Tumor (CD21+) > CD8 T cell	Vessel > Tumor (CD21+)
4	CD45R0	ICOS	Stromal / Fibroblast	Vessel > CD4 T cell	Tumor > Tumor
5	Ki67	Ki67	Tumor (Podo+)	APC > Stromal / Fibroblast	Stromal / Fibroblast > Tumor (CD21+)
6	HLA-DR	CollagenIV	CD4 T cell	Tumor (CD21+) > Tumor (CD21+)	Tumor (Ki67+) > CD4 T cell
7	CD3e	CD8	Tumor	Tumor (CD21+) > B cell	Tumor (CD15+) > Tumor (CD21+)
8	CD49f	CD3e	Lymph vessel	CD4 T cell > Macrophage	Tumor (CD21+) > B cell
9	PanCK	CD57	Granulocyte	B cell > Naïve immune cell	Tumor (Ki67+) > Tumor
10	PDL1	CD38	Tumor (Ki67+)	Stromal / Fibroblast > Tumor (CD21+)	Tumor (CD20+) > CD4 T cell
11	CD68	CD14	Naive immune cell	APC > B cell	Lymph vessel > Vessel
12	TMEM16A	PDL1	Vessel	Tumor (CD21+) > Lymph vessel	CD4 T cell > Macrophage
13	CD34	CD56	Tumor (CD15+)	B cell > CD4 T cell	Tumor (CD20+) > Tumor
14	aSMA	CD20	CD8 T cell	APC > Granulocyte	FoxP3 @ 80μm
15	CD4	GranzymeB	Macrophage	APC > Macrophage	p16 @ 30μm

Table 3: Top 15 features for the RSF models trained on different feature sets, ranked by permutation importance. Feature sets match the ones listed in main Figure 3.

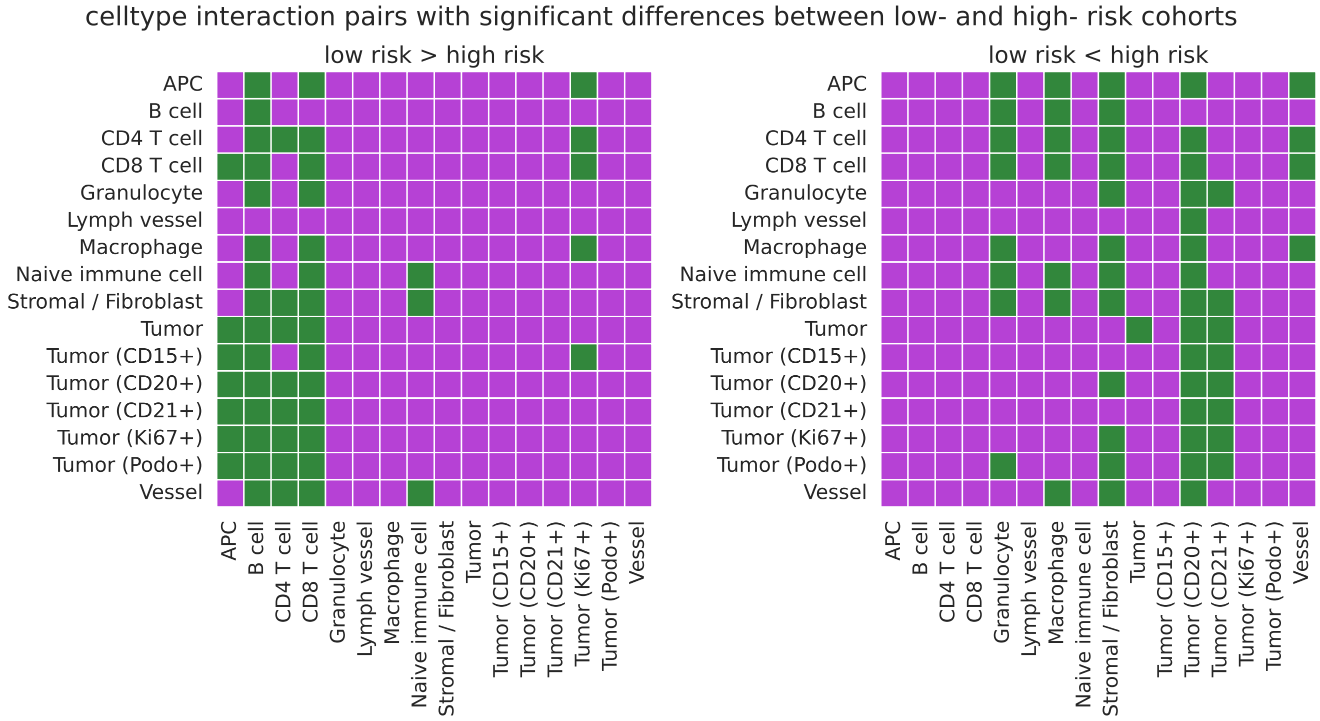

Figure 6: Heatmaps showing cell type interaction pairs with significant differences in their neighborhood fraction values between low- and high-risk cohorts. Neighborhood fraction values are computed using $k = 10$ (Methods). The left heatmap shows significant pairs when values are greater in the low-risk than the high-risk cohort, and vice versa for the right heatmap. Each heatmap element h_{ij} corresponds to neighborhood matrix element m_{ij}, the fraction of celltype j within the k-nearest neighbors of celltype i, averaged across all cells of type i in the sample.

References

[1] Baddeley, A., Rubak, E., and Turner, R. (2015) Spatial point patterns: methodology and applications with R, CRC press, .

[2] Patwa, A., Yamashita, R., Long, J., Risom, T., Angelo, M., Keren, L., and Rubin, D. L. (2021) Multiplexed imaging analysis of the tumor-immune microenvironment reveals predictors of outcome in triple-negative breast cancer. Communications Biology, **4**(1), 1–14.

[3] Ishwaran, H., Kogalur, U. B., Blackstone, E. H., and Lauer, M. S. (2008) Random survival forests. The annals of applied statistics, **2**(3), 841–860.

[4] Pölsterl, S. (2020) scikit-survival: A Library for Time-to-Event Analysis Built on Top of scikit-learn. Journal of Machine Learning Research, **21**(212), 1–6.

[5] Harrell Jr, F. E., Lee, K. L., and Mark, D. B. (1996) Multivariable prognostic models: issues in developing models, evaluating assumptions and adequacy, and measuring and reducing errors. Statistics in medicine, **15**(4), 361–387.
